# A Systematic Review on the Research Progress and Evolving Trends of Occupational Health and Safety Management: A Bibliometric Analysis of Mapping Knowledge Domains

**DOI:** 10.3389/fpubh.2020.00081

**Published:** 2020-04-02

**Authors:** Yujie Wang, Hong Chen, Bei Liu, Menghua Yang, Qianyi Long

**Affiliations:** ^1^School of Management, China University of Mining and Technology, Xuzhou, China; ^2^De Anza College, Cupertino, CA, United States

**Keywords:** occupational health and safety management, theme evolution, bibliometric analysis, mapping knowledge domains analysis, review

## Abstract

**Background:** Effective occupational health and safety management (OHSM) is important to employee health, enterprise sustainability, and social stability. However, scientific research into OHSM lags behind practice, and contextualizing OHSM research from the perspective of its historical evolution is urgently required.

**Methods:** The mapping of knowledge domains, based on bibliometric analysis, was adopted to classify 4,852 journal articles related to OHSM listed in the core database of Web of Science and published between 1900 and 2018.

**Results and Conclusions:** Risk assessment/management at the organizational level were found to have always been research hotspots, and the relationship between safety culture/atmosphere, sickness absence, and safety performance, among other factors, have become core research topics in the field in recent years. Research at the individual level has gradually evolved from an early focus on physiological problems such as work-related musculoskeletal disorders and low back pain, further toward issues such as occupational stress, mental health, and quality of life. In addition, the results of clustering analysis based on high-frequency keywords indicated six aspects of OHSM: OHSM mode and method; OHSM systems and standards; OHS risk assessment and management; OHSM and safety culture; mental health and quality of life; and specific disease management. Future development trends in OHSM research are described providing theoretical and practical reference for further study.

## Introduction

Occupational health and safety (OHS) has become a major issue that countries face globally, with solutions to enhance OHS urgently required in the modern industry. According to a preliminary estimation by the International Labor Organization, approximately one employee dies from an occupational accident or occupational diseases every 15 s, and ~2.34 million employees die from work-related diseases every year. Of these, 86.3% die from occupational diseases, and these numbers continue to increase (1). Economic losses caused by production safety accidents and occupational diseases are huge every year, amounting to ~US$3.3 trillion, which accounts for about 4% of the world's gross domestic product (2). At the same time, with the acceleration of the process of global economic integration and the liberalization of trade and investment worldwide, as well as the application of new technologies, new materials, and new processes in modern industries, many countries are beginning to face the dual challenges of traditional and modern occupational hazards (3). On the one hand, traditional occupational diseases such as coal worker pneumoconiosis are still the “difficult OHS problems” in the United States (US) and other countries (4, 5). On the other hand, with the intensification of enterprise competition and the acceleration of life pace, symptoms of chronic pain (6), mental weakness, job burnout (7), and depression (8) perceived by individual employees in the workplace are increasingly serious. These considerable burdens in OHS not only put forward greater requirements in management practice, but also make it more urgent to grasp the historical context of OHS management (OHSM). Therefore, answers to the following questions are necessary: which changes have appeared on the maps of OHSM, given social and economic development? What were the main research themes at different stages? Given these factors, what will the evolutionary tendency be?

Scholars have carried out relevant studies on health and health issues in the workplace from different perspectives. In these studies, researchers mostly discuss from the perspective of the importance of developing OHSM as part of the sustainable development of enterprises and the improvement of OHS to improve the competitiveness of enterprises (9). A few studies have discussed changing trends in OHSM; for example, Guldenmund reviewed relevant literature on safety culture and atmosphere published between 1800 and 2000 from the perspective of social and organizational psychology, pointing out that safety culture and atmosphere can be used as measures of organizational safety performance (10). Cohen and Kunreuther reviewed the articles on risk management published by Kleindorfer, concluding that risk management is the key point of OHSM (11). Robson et al. attempted to review the effectiveness of OHSMS, based on 13 articles in the Journal of Safety, Medicine and Public Policy (12). Floyde et al. reviewed the OHS challenges faced by small and medium-sized enterprises and attempted to review the importance of OHS training and e-learning for improving OHSM (13). Fan et al. collected 128 articles on OHS-related topics from seven top journals in the discipline of organization management in an attempt to examine OHS issues in business management (14). However, most of the research focused on OHS at a micromanagement level, with few systematically and comprehensively literature review on OHSM (15), in which a lack of research persists and with research failing to inform on research hotspots and evolutionary processes based on an evolutionary perspective.

To comprehensively and systematically understand the research status of and development trends in OHSM, we combined a literature review with mapping of knowledge domains methods to examine development and future trends in OHSM by mining previous research results. On the one hand, we outline the systematic context of OHSM research, connecting classic and emerging OHSM research hotspots, and expanding the theoretical cognition of research in this field. On the other hand, we provide important reference values for organizations to reasonably plan OHSM system effectively to promote improvement in the physical and mental health of employees.

## Process and Methods

### Methodology

The mapping of knowledge domains is a research method that organically integrates traditional literature metrology methods with modern text mining and complex network, mathematics, statistics, and computer science methods and visualization technologies, to comprehensively analyse the development of science. Mapping knowledge domains belongs in the field of scientometrics and can not only be used to extract the frontier, hotspot, and evolutionary trends of specified fields of research, but also for panoramic reproduction of research in a field, using a unique visualization effect (16). Therefore, it has received extensive attention from researchers in the fields of bibliometrics, scientometrics, information science, and information visualization (17). Specific methods include citation analysis, co-word analysis, cluster analysis, word frequency analysis, and social network analysis. In this paper, co-word analysis, cluster analysis, and other methods will be used to analyze the retrieved literature.

Co-word analysis is one of the most commonly used methods in bibliometrics as part of content analysis, and is both a quantitative and qualitative method (18). It can be used to study the research focus and structure of a certain discipline, based on the following two points. First, research hotspots of interest to scholars are not single and isolated, but composed of a series of keywords or subject words closely related in content. Second, irrespective of the differences in the social and knowledge backgrounds of researchers, the subject vocabulary they use is basically the same when considering the same research hotspot. Compared with other methods such as co-citation, co-word analysis is not only more applicable to mature disciplines, but can also be applied to the research hotspots, knowledge hotspots, and development trends of emerging disciplines. Therefore, it has been widely applied to research hotspots and development trends in artificial intelligence, scientometrics, information science, and many other fields.

Cluster analysis is a method of data classification that does classify information according to degree of similarity degree. By transforming text into word vectors and marking entries with different weight values, we can calculate the degree of similarity (affinity or dis-affinity) between measurement samples or indexes statistically and cluster variables with a large degree of similarity into a class based on these statistics. Other variables that are more similar to each other in other categories are all aggregated and the different types separated one by one to form a classification system, from small to large. Finally, the whole classification system is drawn as a pedigree graph to represent the affinity of all the variables. It is an important tool for knowledge discovery and data mining, having played an important role in recent years in text mining in research on organization management (19), work time (20), safety science (21), fracking science (22), and other fields.

Based on this, we introduce concepts of scientific econometric analysis such as co-word analysis, social network analysis, and cluster analysis, into the process of sorting and summarizing OHSM-related research, and explore the current main OHSM research categories and their relationships with each other.

### Data Collection and Data Processing

**Figure 1:**
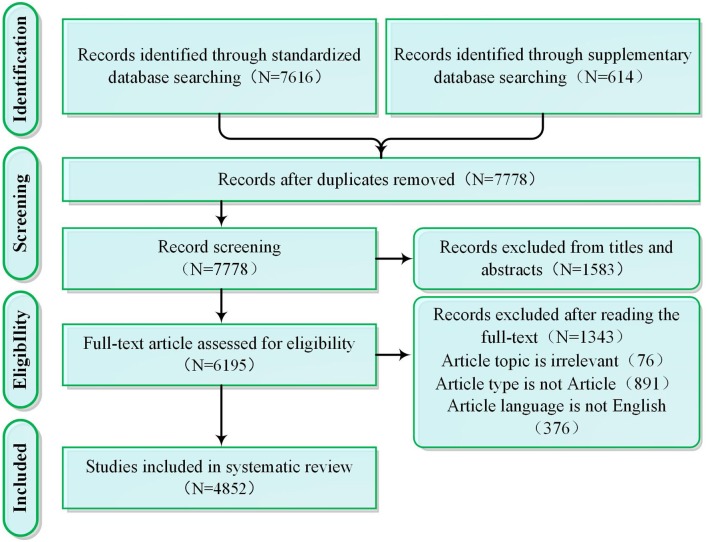
PRISMA diagram.

Choosing the right database is a key step in the bibliometric analysis. As for database type, Web of Science (WoS) is a typical citation database, which contains literature abstracts and other data such as citation information, facilitating for bibliometric analysis (23). As for the scope of inclusion, the database included in WOS is in the direction of natural science and social science, covering a large literature related to OHSM (24). As for data quality, WoS covers a large number of a reliable and high-quality data sources in natural and social sciences (25, 26). Therefore, based on the research purpose of clarifying the research status and development venation of OHSM, we firstly chose WoS rather than all databases, which has a wide range of data, high data quality, and citation information to facilitate literature bibliometric. We set the time range of publishing to 1900–2018 for retrieval. To ensure adequate recall of retrieved data, we did not set specific research categories for article retrieval. In terms of keyword selection, various terms are frequently used in OHSM research, including “occupational health management,” “occupational safety management,” and “occupational health and safety management,” with “OHS management,” “OSH management,” and some other categories used less frequently. To obtain the most extensive information, and according to the retrieval and matching rules of phrases in Web of Science, we adopted the classical form of expression about OHSM, namely “occupational health management,” “occupational safety management,” “OHS management,” and “OHS management” as the retrieval words. After eliminating the duplicated items, irrelevant items, non-article items, non-English items, etc. (see Figure 1), 4,852 journal articles were included in the systematic review.

**Figure 2:**
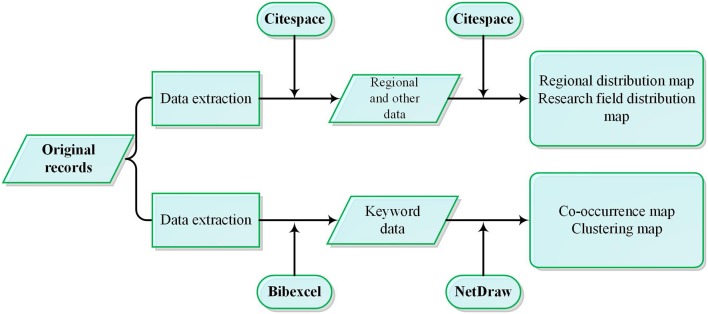
Data processing flowchart.

BibExcel software was used for data extraction and bibliometrics, and CiteSpace, NetDraw, and other software were used to draw the visual knowledge map (The specific data processing operation flowchart is shown in Figure 2). The main functions of BibExcel—scientometrics research software developed by Persson, a Swedish scientometrician—include bibliometric analysis, citation analysis, co-citation analysis, and cluster analysis and thus the software was suitable for data extraction in this study. NetDraw is representative social network analysis software developed by Steve Borgatti of the School of Business and Economics, Gatton State University, Kentucky, US. Due to its simplicity and strong visualization function, NetDraw has been widely used in research involving social network analysis (27). CiteSpace is an information visualization tool specially developed by Chen Chaomei from Drexel University in the US for the analysis of academic literature. It is suitable for multi-element, time-sharing, and dynamic complex network analysis, and can detect hot topics and their evolution in a certain discipline or field (17, 28). CiteSpace has been widely used for detecting and analyzing the output distribution of research topics (29), changing trends in research frontiers, and the relationships between different research fronts (30).

CiteSpace uses node names, node sizes, node ring colors, etc. to present the information contained in the sample data. For example, in the regional distribution map generated by CiteSpace, the nodes represented each named region, the size of the nodes represents the number of articles (the larger the node, the more the number of articles), the pink outer ring of the node represents the centrality (the larger the pink ring, the higher the centrality, indicating that the region has a greater influence in the network structure), and the connection describes country cooperation (the more densely connected the region, the more international cooperation in the region).

NetDraw presents the information contained in the sample data through the color patch area and connecting lines, etc. For example, in the keyword co-occurrence map generated by NetDraw, the color patch area of a keyword is proportional to its frequency of citation. That is, the larger the color patch Keywords, the more frequently they are cited, and the lines in the map indicate the co-occurrence relationship between keywords. The denser the lines, the closer the keyword is to other keywords, and the greater the role it plays.

When drawing co-occurrence maps and cluster maps, it is necessary to screen the extracted keywords to extract high-frequency keywords in the field. We adopted the calculation formula for the number of high-frequency words according to Rosenberg (31).

(1) $$\begin{array}{l} N=\frac{1}{2}\left(-1\pm \sqrt{1+8I_{1}}\right) \end{array}$$

In Equation (1): *N* represents the number of high-frequency keywords, *I*_1_ represents the number of keywords that occurred only once.

## Results

### Spatiotemporal Knowledge Map and Its Analysis

#### Time Distribution Map of OHSM Research

Detailed contents including the number, publication regional, and research field of each OHSM literature publication were identified, to enable a comprehensive grasp of its overall research status and trends. We compiled statistics on literature published from 1900 to 2018, and the annual changing trends are shown in Figure 1.

**Figure 3:**
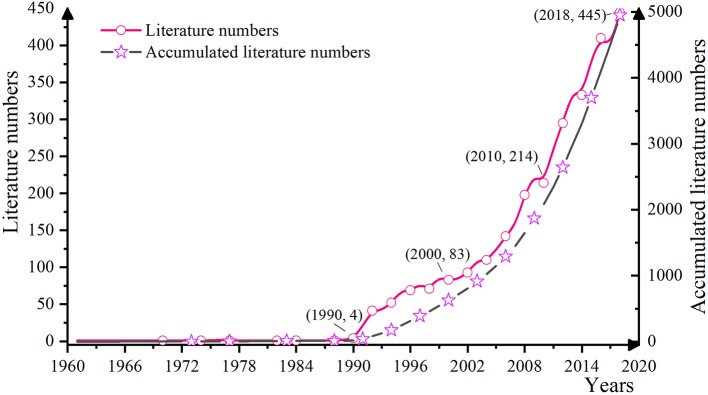
Annual number distribution of selected articles related to occupational health and safety management.

As can be seen from Figure 3, there was an obvious trend to an increasing number of OHSM publications, shown by a steady rise. From the first OHSM literature published in 1961 (32) to the 1990s, the number of OHSM publications published each year showed almost zero growth. From 1990, the number of research papers on OHSM began to grow exponentially, and this point in time can be regarded as the starting point of the OHSM research boom. Since the beginning of the twenty-first century, research in this field has increased dramatically. After 2010, the annual average number of articles published reached 258, reaching a peak in 2018 (445 articles). At this point, it is necessary to summarize the previous research hotspots, explore new hotspots and breakthroughs in combination with development trends in related fields, and prepare for further in-depth research.

#### Spatial Distribution Map of OHSM Research

**Figure 4:**
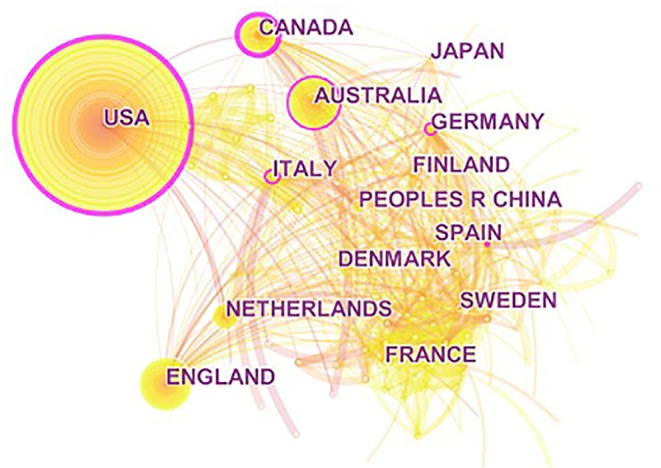
Regional distribution of occupational health and safety management research.

To investigate the regional distribution of relevant OHSM research and international cooperation, CiteSpace was used to generate regional distribution maps (Figure 4). Looking at the map as a whole, there were 132 nodes and 724 links in the national and regional cooperation network map, and the overall density of the network was only 0.0837, indicating a scattered research area, with low cooperation among authors from different countries and regions; From the size of the nodes, it can be seen that the regional distribution of OHSM related research showed a multi-polarization trend, mainly concentrated in North America, Europe, and Australia, etc., among which the United States had the highest contribution rate of literature (*n* = 1,290), followed by the United Kingdom, Australia, Canada, Netherlands, Italy, Germany, etc. From the centrality of each node, the United States had the highest degree of centrality (centrality = 0.34), followed by Canada, Australia, Italy, and other countries, indicating that these countries have more cooperation and interaction with other countries in the field of OSHM research.

**Figure 5:**
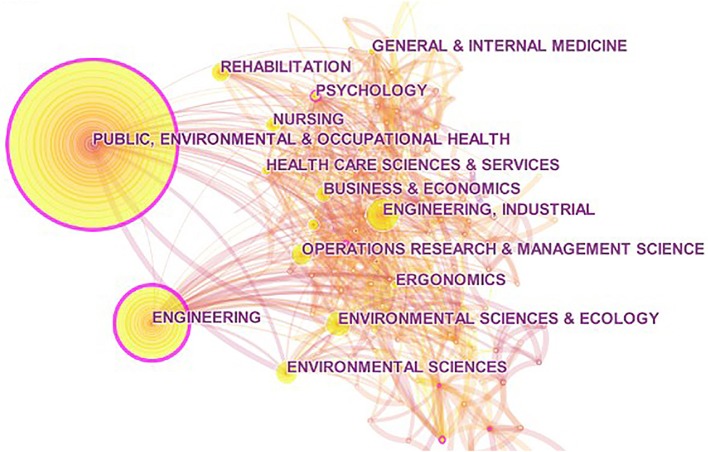
Research field distribution of occupational health and safety management.

We also used the co-occurrence analysis function of CiteSpace to generate a distribution map for the research field of OHSM (Figure 5), to reveal the relationships between major disciplines in this field. Shown in Figure 5, the research field was divided into three fields. One was “industrial-environment-health” including “*public, environment & occupational health*” (*n* = 1,684), “*engineering*,” “*environmental science & ecology*,” and “*environmental science*.” Another field focused on the “diagnosis, rehabilitation and health care” of occupational diseases, including “*rehabilitation*,” “*psychology*,” “*pathology*,” and “*pharmacology*.” In addition to these two research centers, there were also studies on business and economics, such as “*management science*” (*n* = 315) and “*business and economics*” (*n* = 269).

**Figure 6:**
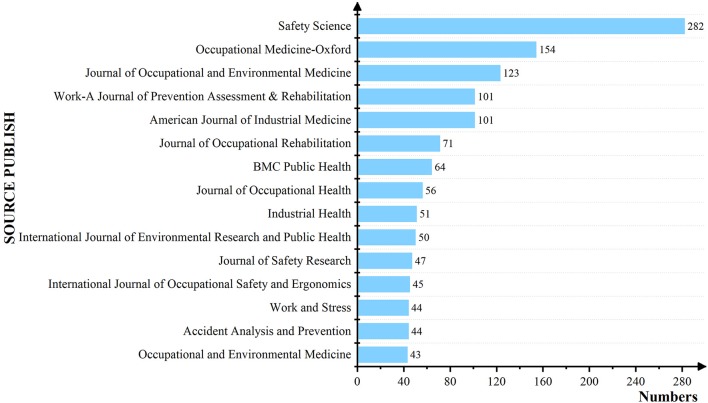
List of 15 Journals that have published at least forty research articles.

We also counted the papers published on OHSM by various journals, to identify the core publication in OHSM research (Figure 6). The number of published journals on related topics was high, and so only high-frequency publication journals are shown, in Figure 6. In terms of journal type, most journals publishing OHSM-related topics concentrated on occupational health, medicine, management, psychology, and related fields. Among them, the Journal of Safety Science ranked highest for number of publications (*n* = 282).

### Content Knowledge Map and Its Analysis

#### Co-Occurrence Map of OHSM High-Frequency Keywords

To sort out the research directions and hotspots in the field of OHSM, the keyword co-occurrence map was constructed using keyword co-occurrence technology. According to the BibExcel processing results, after the processing of singular and plural terms and synonyms, a total of 8,817 original English keywords were obtained from 4,852 journal articles. These were used as the basis for analyzing the OHSM research. According to the high-frequency keywords calculation formula (Equation 1), we calculated that there are 116 high-frequency keywords in the 4,852 journal articles. Due to space limitations, we only listed the 20 highest-frequency words (Table 1).

**Table 1:** Top 20 high-frequency keywords related to OSHM.

**Rank**	**Keywords**	**Frequency**	**Rank**	**Keywords**	**Frequency**
1	Occupational stress	290	11	Safety culture	83
2	Workplace	138	12	Mental health	71
3	Risk management	136	13	Return to work	68
4	Prevention	130	14	Safety climate	61
5	Risk assessment	129	15	Quality of life	58
6	Musculoskeletal disorders	128	16	Job satisfaction	56
7	Intervention	125	17	Low back pain	53
8	Rehabilitation	108	18	Occupational stress management	50
9	Occupational therapy	102	19	Health promotion	48
10	Occupational exposure	92	20	Intervention	48

*The keywords Occupational health and safety (n = 690) and Occupational health and safety management (n = 274) have been removed from the above table*.

**Figure 7:**
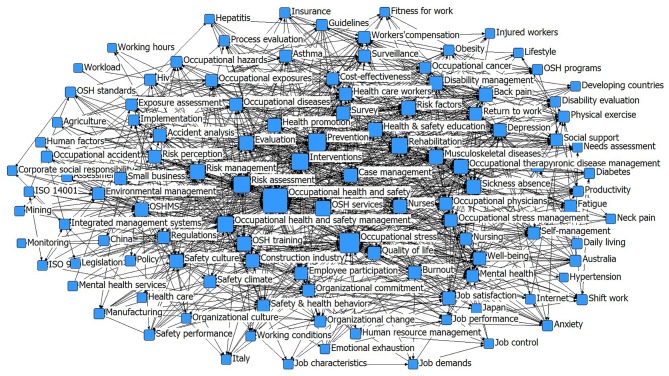
Co-occurrence network map of high-frequency keywords.

After selecting the high-frequency keywords, the co-occurrence matrix was constructed using the BibExcel keyword number results, and by importing the co-occurrence matrix into Netdraw software, a social network diagram of high-frequency keywords in the field of OSHM was obtained (Figure 7). The graph objectively presents research hotspots in the field of OHSM in a multidimensional, simplified, and visualized way. As shown in Figure 7, occupational stress, risk management, risk assessment, mental health, and management system were the core hot words in OHSM research. Among them, “occupational stress” (*n* = 290), “risk management” (*n* = 136), “prevention” (*n* = 130), “risk assessment” (*n* = 129), “safety culture” (*n* = 83), “mental health” (*n* = 71), and “quality of life” (*n* = 58) occur frequently, indicating the importance of these words and the foundation provided by them. “Risk management,” “risk assessment,” and “mental health” had high centrality, indicating that these words were closely related to other words.

**Figure 8:**
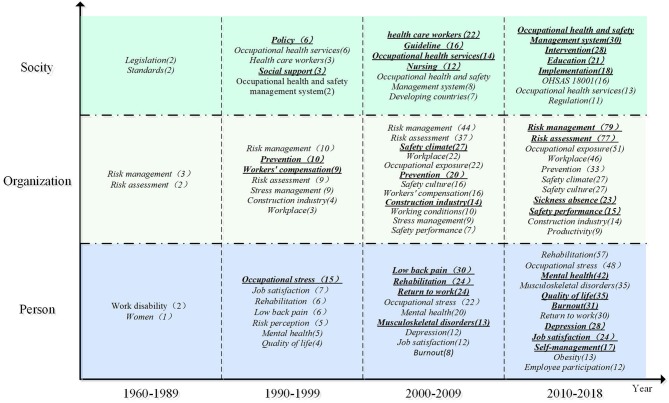
Time zone distribution of high-frequency keywords.

OHSM is related to the health of employees, the sustainable development of enterprises, and social stability. Most of the existing research is studied from these three levels. Based on this, we analyzed the historical literature in the field of OHSM from the perspective of individual–organization–social integration (Figure 8), with the intention of classifying and presenting the content of the research, and outlining it according to time clues. From this, we were able to control the evolution of the knowledge map in the time dimension, and intuitively understand the layout characteristics of keywords in different time periods. This enabled us to view the dynamic processes affecting OHSM research hotspots between time points and from the whole to the local.

Figure 8 shows the evolving trends in OHSM research hotspots. As can be seen from the figure, as time goes by, the keyword layout continues to change. Due to the small number of studies on OHSM before the 1990s, pre-1990 articles were grouped into one time period. The results show that research at the individual level mainly focused on the work experience of employees (occupational stress, mental health, job satisfaction, quality of life, etc.), and the management of specific diseases such as low back pain (LBP). Research at the organizational level mainly focused on health risk management, risk assessment, safety culture, and safety atmosphere. At the social level, the research mainly focused on OHSM systems and standards, OHS services, and other related aspects. From the perspective of time frame, the related topics showed developing trends in richness, and the hotspots were observed to have changed significantly from around 1990. A previous focus on disease management had evolved to focusing on the work experience of employees, such as occupational stress, mental health, quality of life, risk perception, and other issues, and exploration of these new research topics continues to this day. After 2000, LBP, rehabilitation, occupational stress, mental health, and musculoskeletal disease at an individual level and related topics began to emerge in large numbers; corresponding topics of safety climate, safety culture, prevention, construction industry, and related research at organizational level also gradually increased in focus. Health services, care workers, and other research at a social level gradually became hot topics. After 2010, mental health, quality of life, job burnout, depression, job satisfaction, self-management, employee participation, safety performance, OHS education, OHS intervention, and OHSMSs became new focuses. These changes reflect the changing focus of OHSM during its advancement and development.

#### Cluster Map of OHSM High-Frequency Keywords

**Figure 9:**
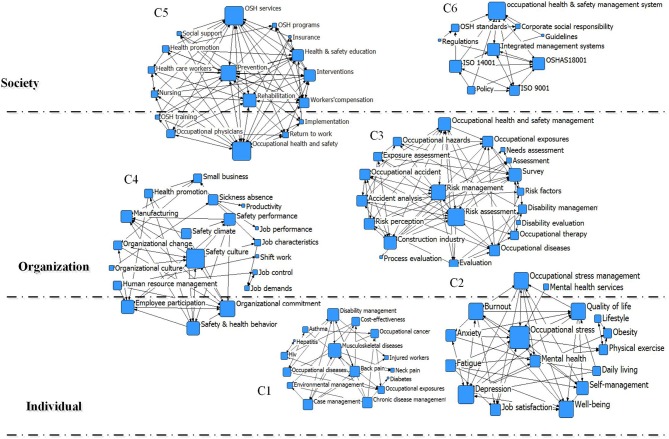
Clustering map of high-frequency keywords.

To further study the topic structure of OHSM-related literature, we conducted cluster analysis of high-frequency keywords based on the clustering function of the CiteSpace and NetDraw software. After processing the clustering results, a cluster distribution diagram was drawn (Figure 9).

After excluding keywords that are not strongly related to the topic, we divided the keywords into six clusters: specific disease management (C1 on Figure 7); occupational stress, mental health and quality of life (C2); OHS risk assessment management (C3); OHSM and work elements such as safety culture (C4); OHSM model and methods (C5); and OHSM systems and standards (C6). According to the results, occupational stress, mental health, and quality of life at the individual level; OHS risk assessment and management at the organizational level; OHSM and work elements; OHSMS standards, OHS prevention, and rehabilitation at the social level were hotspots in OHSM research. Among them, occupational stress, risk assessment, safety culture/atmosphere, and OHSMS had a high centrality, which on the one hand shows the importance of these terms and indicates the foundation they provide, and on the other hand shows that OHSM is a comprehensive governance process that takes into account the micro, medium, and macro. Based on an integration perspective stratified at individual–organization–society levels, the location of each cluster was identified in the hierarchy, with cross-level clustering variables in the C2, C4, and C5 clusters. In the actual research process, the clusters were not absolutely independent from each other, and we adopted a method of expressing this to enable readers to clearly understand the distribution properties of the current OHSM research.

## Discussion

### Evolving Trends Under Timeline

A clear trend of a steady increase in the number of studies published on OHSM was seen. The evolution of OHSM research could be divided into four stages: “infancy” (1960–1989), “break out” (1990–1999), “rapid growth” (2000–2009), and “multiple development” (2010–present). From a developmental trend perspective, research on OHSM remains a major topic and has a broad potential for application in the future.

During the infancy period, there were few studies on OHSM, and research contents were mainly limited to OHS programs and risk assessment management. The reason is that in the 1960s, due to the rising prevalence of chronic diseases in the US and the sharp rise in medical costs, the US insurance industry took the lead by proposing the concept of health management, which was widely embraced by both the government and private enterprises. Many US enterprises successively launched employee assistance programs, and these have since gradually extended to occupational health promotion programs (1980–present) and employee enhancement programs (1988–present). The prevalence of these Programs has enabled scholars to actively explore OSH programs. In addition, with the promulgation of the Occupational Health and Safety Act of 1970 (33), the US Centers for Disease Control and Prevention (CDC) authorized the Michigan Health Management Research Center to conduct research related to employee health management. They developed health risk assessment tools and health management methods based on prospective medical techniques, and promoted the use of health management systems in the US. At this time, there was some degree of theoretical basis and some practical operational methods within OHSM, and these began to be applied, developing rapidly within enterprises (34). OHS risk management research began to rise, becoming the focus of long-term attention in the field of OHSM.

During the breakout period, research into OHSM began to pour in. Occupational stress and occupational stress management became core issues in OHSM research, the reason is that evidence from many aspects indicates that a growing number of workers to be victims of working pressure (35). Employees' work efficiency was declining due to increasing work pressure, a phenomenon that directly or indirectly affects the employer's revenue and profit. To improve production efficiency and obtain greater profits, the employer needs to consciously meet the psychological needs of employees, to prevent possible health problems (36), through stress management plans (37), and the provision of health management services (38). At the same time, health management policy research received extensive attention from scholars as with the acceleration of international integration, OHSM issues, which are closely related to the production process, had attracted the attention of the international community. In addition to the US, some European countries, including the United Kingdom, Germany, and Finland, were beginning to explore OHSM, with relevant legislation and policies continuously introduced and improved, and different forms of health management organization gradually being established (39). In 1999, OHSMS requirements (OHSAS18001) were jointly issued by the British Standards Institution and 13 other major global standard-setting bodies, certification bodies, and professional organizations integrating many safety management systems. Since then, the standards have been widely used in many developed countries, and research into this OHSMS and its standards has become an increasing focus of research.

During the rapid growth period, research into OHSM gradually deepened. Studies on chronic occupational diseases such as occupational LBP, work-related musculoskeletal disease (WMSD), and occupational asthma at the individual level emerged in large numbers, mainly due to the increasingly prominent problems such as WMSD and LBP, which have become the most common occupational diseases in many industrialized countries. These conditions seriously affect the health and efficiency of workers, causing serious economic losses and arousing widespread concern from government and health research institutions. A large number of studies on the distribution characteristics, influencing factors, treatment, rehabilitation, and prevention of these occupational diseases have been conducted (40). At the same time, the safety atmosphere and culture, work environment, occupational exposure, and other work elements from the organizational perspective entered the core topic area of OHSM research, with scholars beginning to realize that the perception of a safety culture by employees in the workplace can influence and reduce unsafe behaviors and the occurrence of workplace exposure events (41), thus improving the safety performance and work output of enterprises (10, 42). Research on OHS guidance, OHS service, OHS care, and other health management models and methods from the social perspective also attracted extensive attention from scholars during this period, mainly due to the increasing prominence of occupational diseases such as WMSD and LBP. To solve these problems, many countries started to issue OHS guidelines (43) and train health care workers in OHS (44). In addition, some researchers actively explored the OHSM model. For example, Musich et al. (45) proposed three categories of health management model: health promotion and prevention; acute treatment; and disease management, in which examination and prevention are key in the early stage; treatment and recovery as important in the middle stage; and lifestyle interventions are reflected in all aspects of healthy living (45).

During the rapid growth period, OHSM research became further enriched and involved more comprehensive content. At the individual level, research focused on occupational stress, mental health, quality of life, and job satisfaction. Researchers actively explored the influencing factors (46) and influencing effects (47) of, and interventions for (48), mental health problems such as burnout and depression. Quality of life (QOL) is an extension of mental health that comprehensively reflects the physiological, psychological, spiritual, and social state of a person. With the advent of the era of knowledge economy and an awakening of employees' health awareness, QOL has attracted increasing attention and became increasingly included as an aspect of OHSM for research. For example, Hoon et al. (49) studied the relationship between the level of self-health management and the QOL of people with chronic musculoskeletal diseases. Organizational level is the research focus of risk assessment and management, sickness absence, and organizational performance, the reason is that the OSH management is more and more regarded as an important part of enterprise sustainable development. Additionally, OHSM level is closely related to organizational performance and organizational commitment, and OHS risk assessment management is key to OHSM of an enterprise. Research on the OHSMS, OHS education, OHS intervention, and OHS implementation from the social perspective has also been a focus of attention in recent years. For example, Floyde et al. reviewed the OHS challenges faced by small and medium-sized enterprises and believed that training and education of employees through knowledge management and e-learning were the key to dealing with them (13).

### Evolving Trends in Spatial Distribution

As for the distribution of research regional, the research on OHSM was mainly concentrated in countries such as the United States, The United Kingdom, and Australia, while China and other countries had fewer studies on OHSM, and had less cooperation and interaction with other countries in this field. This could be caused by several reasons: (a) The United States and other developed countries have paid more attention to employee health and safety, and related research has started earlier (50). (b) China and other countries started research on OHSM relatively late and paid less attention to safe production and healthy production in the past (51). (c) China and other developing countries lack research institutions and leading figures with sufficient influence in OHSM, restricting research output on OHSM. However, with the implementation of health strategies in developing countries such as China, OHSM research has also gradually flourished. From 2010 to 2018, the average annual growth rate of relevant articles in China was 19.37%, far higher than that of other developing countries, indicating China and other countries will play an increasingly important role in OHSM research. In the future, macro-guidance and the establishment of a long-term guarantee mechanism are required to promote cooperation among authors at scientific research institutions in different regions, thereby achieving the goal of improving the level of OHSM.

As for the research fields, the research on OHSM was mainly distributed in the fields such as “industry–environment–occupational health,” and “diagnosis–rehabilitation–health care.” This is mainly because the health and health problems of employees are closely related to their working environment, workflow, health protection, and other external conditions (14). And the protection measures for employees' health and safety mainly include inspection and prevention in early-term, treatment, and recovery in mid-term, lifestyle intervention in full cycle, etc. (45, 52). Besides, the health and safety of employees is an important guarantee for the sustainable development of the enterprises' economy, so the research on OHSM also appears in many fields such as “business and economics.”

As for the distribution of journals, the research on OHSM was mainly published in medicine, sociology, psychology and management, and other related academic journals. This is mainly due to the fact that OHSM is a comprehensive problem that takes medicine, sociology, management, and other disciplines into consideration. Therefore, the research on OHSM is mainly focused on the different kinds of journals above. Among them, the supports of the Safety Science journal on OHSM research is greatly, and its volume of publications is far more than other journals. This is mainly because: (a) The journal is an interdisciplinary international academic journal of safety, and the articles submitted to the journal can be social science, management, natural science, and engineering technology (53). (b) The main papers' topic of this journal covers a wide range, including physical and engineering aspects of safety, social and policy aspects, organizational characteristics, etc. It also covers risk management, effectiveness of safety measures, safety standardization, safety laws and regulations, safety inspection, insurance, safety cost, behavior, etc. (54). (c) This journal is highly professional, influential (Q1) and has a large number of articles. Many scholars are inclined to contribute to this journal (55). Therefore, the amount of contributions of the Safety Science journal to OHSM is far more than other journals.

### Development Trends in Theme Distribution

The key words co-occurrence and cluster maps indicated that research perspectives on OHSM have gradually diversified, and could be divided into the following six themes: OHSM mode and method; OHSM systems and standards; OHS risk assessment and management; OHSM and safety culture; mental health and quality of life; and disease-specific management. Further integration of the six clusters can be roughly divided into three processes: theoretical level, operational level, and application level.

#### OHSM Mode and Method

The study of OHSM mode and method focuses on how to mitigate the negative impact of OHS problems and explores effective OHSM models and methods. Core keywords included OHS programs, OHS services, prevention, rehabilitation, and interventions. An early OHS management mode was medical diagnosis and treatment for a specific disease (32). When disease is found in employees, enterprises need to invest in helping employees with treatment, to reduce the burden on them. OHSM should continue throughout medical diagnosis and treatment (56). Employee assistance programs, occupational health promotion programs, and employee enhancement program can be gradually extended on the basis of disease management. In response to relevant laws and regulations, many researchers carried out theoretical and practical research into the prevalence, implementation cost, and implementation effects of an OHS program (57). At the same time, OHSM modes have gradually changed in emphasis from post-treatment and diagnosis to early detection and prevention, interim treatment and recovery, and whole-cycle lifestyle intervention. Musich et al. proposed that health management should be divided into three aspects: health promotion and prevention; acute treatment; and disease management (45). OHSM modes are currently developing a trend toward overall management, all-staff management, sustainability, and dynamic tracking. When employees are in a healthy state, enterprises should take certain intervention measures (such as OHS education, OHS training, and OHS knowledge management) and provide certain OHS services (such as regular health examination services.) in readiness for possible OHS challenges (13). Enterprises should be willing to assist in medical treatment, carry out rehabilitation management (52), and provide compensation for employees (58) in the case of occupational diseases. Models and methods of comprehensive health management (59), employee participation management, electronic information intervention, OHS programs, and individualized health management will continue to be a focus for research.

#### OHSM Systems and Standards

Research into OHSM systems and standards highlights the importance of adopting management systems and standards to improve OHSM. Key terms include integrated management systems, OHSAS 18001 (OHSMS requirements), ISO9001 (quality management system), ISO14001 (environmental management system), and standards. With the acceleration of the process of international integration, OHS, which is closely related to the production process, has attracted more and more international attention. In 1999, OHSAS18001 was jointly issued by the British Standards Institution and 13 other major global standard-setting bodies, certification bodies, and professional organizations, integrating many health and health management systems. The main focus of this system is to realize the virtuous cycle of P–D–C–A (Plan–Do–Check–Action), to comprehensively and effectively promote the health and health management of enterprises. Since then, this standard has been widely applied in many developed countries. By 2009, 56,250 facilities in 116 countries had obtained OHSAS 18001 certification (60); many multinational companies (e.g., Apple, Boeing, HP, Coca-Cola.) have obtained OHSAS 18001 management system certification and require their suppliers to obtain certification. The prevalence of the adoption of OHSAS 18001 has aroused the interest of researchers in the integration of OHSMSs with ISO 9000 and ISO 14000 (61). Many studies have shown that the implementation of an OHSMS has a positive impact on corporate security performance, economic and financial performance, and corporate competitiveness (62). Simon et al. further pointed out that integrated management systems can improve corporate coordination, reduce the administrative burden, improve health and health, and ultimately improve efficiency and productivity (63). Some researchers have found that OHSMS certification mainly exists to achieve auditability of OHS performance and meet the needs of external stakeholders, rather than improving health and health (64); however, other studies have confirmed the effectiveness of OHSMS (12, 65). With the prevalence of OHSMSs, many developing countries have promulgated a series of new OHSMSs by referring to international advanced standards and applying them to local conditions. For example, China issued the *Occupational Health and Safety Management System Requirements* and *Occupational Health and Safety Management System Guide* in 2011. With the continuous expansion of OHSMS applications, research on OHSMSs is in the ascendant stage, and the development and application of personalized OHS information systems also show a significant and vigorous development trend.

#### OHS Risk Assessment and Management

This field of research mainly focuses on the development and application of OHS risk assessment and management methods to evaluate the impact of adverse work environments on human health. Its core keywords include risk management, risk assessment, survey, risk perception, exposure assessment, construction industry, and accident analysis. OHS risk assessment at the organizational level has always been a research hotspot in OHSM, having first started in the 1960s. In its early stages, OHS risk assessment research was mainly qualitative. In the 1980s, the US Environmental Protection Agency (EPA) put forward four steps of health risk assessment: data investigation, toxicity assessment, exposure assessment, and risk characterization. Since then, Canada, United Kingdom, the European Union, Australia, and other countries have carried out research into OHS risk assessment, and OHS risk assessment and management related to organizational operations has been of increasing concern to scholars. Joy systematically reviewed changes in the security situation in the Australian mining industry over the previous 15 years, highlighting that improvements of Australia's mine security is largely due to the development and application of risk assessment management methods (66). Joy also asserted that OHS risk assessment management can not only reduce accident rates, but also improve the productivity and financial performance of enterprises. Other researchers have focused on the assessment and management of OHS risks in the construction industry. For example, Haslam et al. analyzed 100 construction accidents and believed that a lack of appropriate risk management was one of the most relevant factors, with 84% of accidents predictable and avoidable if appropriate risk management had been carried out (67). Sousa et al. reviewed studies of accident mechanism, accident analysis, and accident modeling in the field of OHS in the construction industry, considering it necessary to quantify the OHS risks in construction projects and proposing a potential OHS risk assessment model (OHS-PRM) (68). This is mainly because construction is the most dangerous industry in OSH. Construction workers are three times more likely to die on the job than the average worker in all other activities, and twice as likely to be injured. It is noteworthy that at present, OSH risk assessment is relatively lagging behind in developing countries and has not been included in the routine assessment work. However, with improvements in living standards and the strengthening of employee awareness of their health at work, increasing attention has been paid to health risk assessment, and attention to OHS risk assessment and management will continue to increase.

#### OHSM and Safety Culture

Research into OHSM and safety culture investigates the relationship between safety culture/ atmosphere, sickness absence, safety performance, organizational commitment, and other work elements. Core keywords include safety culture, safety climate, safety performance, health and health behavior, sickness absence, and organizational commitment. Research into safety culture incorporates the belief that the health of employees is an important part of human resource management of an organization (69) and that it is a component of enterprise productivity (70). Managers of enterprises must be aware that safety, health, the environment, and traditional enterprise management belong to the same indivisible unity, and that the performance of enterprise is closely related to the health of its employees (14). Promoting an atmosphere of safety and a safety culture improves staff creativity, productivity, and safety performance. Specifically, some studies have found that the perception of a safety culture by employees is conducive to reducing their unsafe behaviors, thereby improving the safety performance and work output of enterprises (10, 42). Safety management has a positive impact on safety performance, economic performance, and financial performance, so protecting the health and health of employees is conducive to improving the competitiveness of enterprises (62). The physical and mental health of employees will influence deviant behavior in the workplace, with employee health management effectively reduce absenteeism, sabotage, workplace violence, and other negative behaviors (71). From this perspective, it is notable that employees with a higher health status tend to have higher job satisfaction (72), higher organizational commitment, and better career development (73, 74).

#### Mental Health and Quality of Life

As well as mental health and QOL, research in this area focuses on occupational stress and other psychological processes perceived by employees as part of the work process. Keywords include occupational stress, mental health, depression, QOL, and well-being. Mental health problems first attracted attention in developed countries, because the increase of work accidents and decrease of work efficiency caused by work pressure or interpersonal relationship problems among employees directly or indirectly affected the income and profit of employers. To improve production efficiency and profits, employers began to consciously meet the psychological needs of employees. Some researchers have studied the causes of mental health problems in employees, pointing out that work pressure caused by the mismatch between work requirements, work control, and employees' own ability will lead to emotional disorders and emotional fatigue, which will then have a negative impact on the physical and mental health of employees (75, 76). Research into employee mental health has also been conducted, including into the negative effects of job burnout, anxiety, and depression, with job burnout often closely associated with absenteeism and high staff turnover rates (77). Burnout also negatively impacts on work performance, damaging relationships and leading to family problems and poor personal health (78). Burnout may also lead to depression (79, 80). Employees with higher mental health tend to have higher job satisfaction (72) and better career development. In term of coping with mental health difficulties, Luthans et al. extended the concept of psychological capital to the field of organization and management, with the potential to lead to a positive organizational behavior psychological state in employees, using interventional measures such as cultivating hope and optimism and promoting self-efficacy to improve the mental health and job performance of employees (81). In addition, some studies have focused on the relationship between mental health and QOL; for example, Amponsah-Tawiah et al. conducted a case study of the mining industry in Ghana, finding that mining equipment, environmental conditions, and work requirements and control were important predictors of QOL and happiness (82). In recent years, with the continuous acceleration of economic globalization, urbanization and informatization, and the increasing work intensity experienced by employees, “white-collar depression,” and other psychological problems seen in developed countries are also emerging in developing countries such as China and India. In developing countries, attention is increasingly being given to the mental health of employees, and this will continue to be a focus of research in psychology and management.

#### Management of Specific Occupational Diseases

Research into occupational disease management mainly focuses on the management of WMSD, LBP, occupational asthma, and occupational pneumoconiosis, as well as other specific diseases. WMSD is caused by muscle, bone, and nervous system injury resulting from adverse factors in the workplace, which has become increasingly prominent and is now the most common occupational disease in industrialized countries such as the US. For the years 1992–2010, WMSD accounted for 29–35% of all occupational injuries and illnesses involving days away from work in the US (83). WMSD has caused widespread concern from government and health research institutes due to its serious impact on the health and efficiency of workers, as well as serious economic losses to organizations and society. The pathogenesis (84), diagnosis and treatment (85), and intervention measures (86) for WMSD have been studied extensively. Occupational LBP is a widespread and serious occupational disease among employees (87), characterized by pain and motor dysfunction. With the continuous acceleration of work pace, LBP has become the most common cause of medical treatment, hospitalization and surgery in the population. The distribution characteristics of LBP (40), influencing factors (88), measurement, and preventive treatment (89) have been studied extensively. In addition, some researchers have focused on other occupational diseases such as occupational asthma. With the continuous advancement of industrialization, the management of WMSD, LBP, occupational asthma, and other occupational diseases will continue to receive extensive attention from researchers.

### Limitations

There are some limitations in this study, which need to be further improved. (1) In terms of data sources, this study selected the WOS database which has a wide range of data, high data quality, and is convenient for bibliometric analysis. Other databases such as PubMed were not included in the systematic study. In the future, data from other databases could be included in the study of OHSM. (2) In terms of research methods, there are many other methods for the systematic review of OHSM research. In this study, we only used methods such as co-word analysis and cluster analysis to analyze the retrieved literature. In the future, citation analysis, meta-analysis, and other analysis methods could be used to study OHSM. (3) In terms of the results of this study, this thesis only used English literature for bibliometric analysis, this search method may lead to deviation in the results of the study (for example, Chinese scholars' related articles in Chinese did not include in the research may lead to some consequences such as lower research centrality on Chinese OHSM), so the applicability of the results needs further research. In the future, data in different languages (Chinese, Japanese, French, etc.) could be included in the OHSM systematic review, further verify the results of this study.

## Research Trends and Conclusions

### Research Trends

#### Development Trends in Time and Space Distribution

In terms of time and space distribution, the amount of research is growing, and research institutions and research field are gradually diversifying. However, unbalanced regional growth, single research institutions, and relatively limited research field are issues in some developing countries such as China. China's exposure to occupational hazards, and the number of new occupational diseases, cumulative number of occupational disease patients, and industrial injury accident death toll are the highest in the world; however, the output and influence of OHSM research are lagging behind. Research on OHSM in developing countries needs to be strengthened, and hopefully with the implementation of improved health strategies, OHSM in developing countries will receive increasing attention from researchers.

#### Development Trends in Research Focus

The research scope of OHSM is constantly expanding and research content is correspondingly enriched. However, certain topics remain a focus of current researchers, especially at the organizational level. OHS risk assessment/management has been one research hotspot. Recent intensification of business competition and the accelerating rhythm of life mean that an increasing number of workers are victims of workplace stress. Employee work efficiency is declining due to increasing work pressure, which directly or indirectly affects the revenue and profit. To improve production efficiency and increase profits, the employer must consciously meet the psychological needs of employees, to prevent possible health problems. In this context, safety culture, mental health, and QOL will receive increasing research focus. In addition, with the increasing prevalence of the use of OHSAS 18001 certification in multinational companies (such as Apple, Boeing, HP, Coca-Cola), research on integrated management systems (including quality management system ISO9000, environmental management system ISO14001, and occupational health and safety management system OHSAS 18001) will become an important focus.

#### Development Trends in Research Logic

From the perspective of research logic, OHSM research continues to extend to the forefront of the health continuum. Early OHS management modes involved medical treatment for a specific disease. Only when employees were diagnosed with disease did the enterprise invest in assisting employees with disease treatment, to reduce their burden. Subsequent employee assistance programs, occupational health promotion programs, and employee enhancement programs are progressive extensions of disease management. OHSM modes have changed from a focus on diagnosis and treatment to early detection and prevention, and mid-term treatment and recovery. OHSM modes have gradually moved toward overall management, all-staff management, sustainability, and dynamic tracking. When employees are in a healthy state, enterprises should provide interventions such as OHS education, OHS training, and knowledge management, and OHS services such as regular health examination, to deal with potential OHS challenges. Where occupational disease occurs, organizations should assist in medical treatment and rehabilitation management, and provide compensation for employees. Early prevention and intervention, treatment and rehabilitation in the middle stage, and late rehabilitation and training will continue to be research focuses.

#### Development Trends in Research Level

OHSM research has shown comprehensive development from the micro level through meso to macro level. Early OHSM research focused on discussion at the enterprise level. With the implementation of a series of OHSMS standards, research has gradually extended to OHS planning, provision of OHS services, the OHS industry, and other aspects at the social level. In recent years, the intensification of enterprise competition, acceleration of life pace, and improvement of employee health awareness have increased OHSM research focus at the individual level; individualized health management, employee self-management, and other related topics are potential research directions.

### Conclusions

This study conducted bibliometric analysis of 4,852 papers in the OHSM field, presenting the research status, evolution process, and main research themes in a visual form to clarify the overall knowledge structure of OHSM research.

(1) Time distribution mapping showed that OHSM research in terms of the number of studies followed a trend in rising volatility. From the perspective of time, the evolution of OHSM could be divided into four stages: infancy (1960–1989), break out (1990–1999), rapid growth (2000–2009), and multiple development (2010–present). After 2006, the number of published research studies showed a rapid growth trend, and OHSM is currently experiencing a multiple development period, thus indicating that research on OHSM offers broad potential.
(2) Spatial distribution mapping analysis showed a trend toward multi-polarization for the research regional and research field. As for the distribution of research regional, the research on OHSM was mainly concentrated in countries such as the United States, The United Kingdom, and Australia. In recent years, research in this area in China and other countries has also gradually flourished. As for the research fields, the research on OSHM was mainly distributed in the fields such as “industry–environment–occupational health,” “diagnosis–rehabilitation–health care,” and “business and economics.” As for the distribution of journals, the research on OSHM was mainly published in medicine, sociology, psychology and management, and other related academic journals. Among them, the journal of Safety Science gave strong support to OHSM research.
(3) Co-occurrence mapping analysis of keywords showed that the theme of OHSM research shows a comprehensive trend that takes into account micro-meso-macro. The terms occupational stress, risk management, prevention, risk assessment, safety culture, mental health, and quality of life were the most topical terms found in OHSM research. Further time frame analysis results showed that OHS risk assessment/management at the organizational level has always been a research hotspot, and research on the relationships between safety culture/atmosphere, absence, safety performance, and other factors has become core in recent years. Research at the individual level has gradually evolved from an early focus on physiological problems such as WMSD and LBP to the deeper topics of occupational stress, mental health, and QOL. Social research as a topic in OHSM is mainly focusing on OHS management system/standard construction and OHS management modes.
(4) Keyword cluster mapping analysis showed that the research scope of OHSM is constantly expanding and research content is constantly enriched, gradually evolving multiple aspects, defined as specific disease management; mental health and quality of life; OHS risk assessment and management; OHSM and safety culture; OHSM mode and method; and OHSM systems and standards. Further integration of the six clusters can be roughly divided into three processes: theoretical level, operational level, and application level.

## Author Contributions

YW and HC: conceptualization and project administration. YW: methodology, software, formal analysis, and writing-original draft preparation. HC, BL, MY, and QL: validation. YW and BL: investigation. HC and QL: resources. MY: data curation. HC, YW, and QL: writing-review and editing. HC: visualization and supervision.

### Conflict of Interest

The authors declare that the research was conducted in the absence of any commercial or financial relationships that could be construed as a potential conflict of interest.
